# High-Density Surface Electromyography Excitation of Prime Movers Across Scapular Positions in the Seated Row

**DOI:** 10.3390/jfmk11010006

**Published:** 2025-12-24

**Authors:** Riccardo Padovan, Emiliano Cè, Stefano Longo, Gianpaolo Tornatore, Fabio Esposito, Giuseppe Coratella

**Affiliations:** Department of Biomedical Sciences for Health, Università Degli Studi di Milano, 20133 Milan, Italy; riccardo.padovan@unimi.it (R.P.); emiliano.ce@unimi.it (E.C.); stefano.longo@unimi.it (S.L.); gianpaolo.tornatore@gmail.com (G.T.); fabio.esposito@unimi.it (F.E.)

**Keywords:** EMG, strength training, pulley exercise, muscle activity, resistance training

## Abstract

**Objectives**: The present study compared the amplitude and spatial distribution of muscle excitation between a seated row performed with a fixed scapular position (fixed-SR) and a free scapular position (free-SR) in resistance-trained men, analyzing concentric and eccentric phases separately using high-density surface EMG (HD-sEMG). **Methods**: Fourteen resistance-trained males (age: 25 ± 4 years; stature: 1.74 ± 0.06 m; body mass: 76.22 ± 5.73 kg) performed fixed-SR and free-SR in a randomized cross-over design using 8-repetition maximum as the load for both variations. HD-sEMG grids recorded the activity from the upper/middle/lower trapezius, latissimus dorsi, lateral/posterior deltoid, biceps brachii, triceps brachii, and erector spinae. Normalized root mean squared (RMS) amplitude and excitation centroids in the mediolateral and craniocaudal planes were computed for the concentric and eccentric phases. Data were analyzed using repeated-measures statistical models, with significance set at *p* < 0.05. **Results**: During the concentric phase, nRMS amplitude was greater for the posterior deltoid in fixed-SR compared with free-SR (effect size [ES] = 0.66), whereas no between-condition difference was observed for the remaining muscles. During the eccentric phase, nRMS amplitude was greater in the fixed-SR for the middle trapezius (ES = 0.67) and the latissimus dorsi (ES = 0.85), with no between-condition differences detected for the remaining muscles. The centroid position analysis revealed that, during the eccentric phase, the middle trapezius centroid was located more laterally in the fixed-SR condition (ES = 0.54), while the posterior deltoid centroid was positioned more caudally in the fixed-SR compared with the free-SR condition (ES = 0.22). **Conclusions**: The fixed-SR and free-SR conditions produce comparable overall muscle excitation patterns, while showing some quantitative and spatial differences in selected upper-back muscles. These results suggest that scapular constraint influences the distribution of muscular excitation rather than overall excitation levels. Accordingly, both variations can be effectively used in resistance training, selecting to fix or free the scapulae depending on the emphasis on the scapular movements rather than a substantial difference in muscle excitation.

## 1. Introduction

Resistance training programs are typically designed to emphasize specific muscles by selecting movements that impose distinct mechanical demands on the musculoskeletal system [1]. Adaptations to resistance training arise from the interaction of neural, molecular, and physiological processes. Early improvements in performance are largely explained by neural factors, including enhanced motor unit recruitment and firing behavior, improved intermuscular coordination, and reduced antagonist co-activation. With continued training, repeated mechanical loading elicits molecular signaling cascades that regulate muscle protein turnover, favoring net protein accretion and supporting hypertrophic remodeling. These processes are accompanied by physiological changes such as altered muscle architecture and tendon properties, as well as enhanced capacity to generate force. At the tissue level, resistance training can increase muscle cross-sectional area through enlargement of individual fibers and remodeling of contractile and non-contractile elements, ultimately contributing to gains in strength and muscle size [2,3,4,5]. Accordingly, analyzing the role of individual muscles during movement execution allows a clearer characterization of the neuromuscular demands imposed by the task [6].

Surface electromyography (sEMG) has been widely used to investigate neuromuscular demands during resistance training, providing insight into how different exercises and technical variations engage specific muscle groups. A substantial body of literature has documented muscle excitation patterns during lower-body exercises such as the squat [7,8], and hip-dominant movement like the deadlift [9,10]. Similarly, upper-body pushing tasks—including multiple bench press variations [11,12,13,14] and overhead pressing variations [15,16,17,18,19],—have been extensively examined. In the context of pulling exercises, previous studies have primarily focused on vertical movements such as lat pull-down variations [20,21,22,23] single-joint tasks like the biceps curl [24,25,26,27] cable-based exercises [28,29] and, to a lesser extent, horizontal pulling movements. Collectively, these studies demonstrate that technical execution can meaningfully influence neuromuscular excitation patterns. Despite the extensive application of sEMG across resistance training exercises, important gaps remain. In particular, horizontal pulling movements such as the seated row have received comparatively less attention, especially with respect to the role of scapular positioning. While seated row exercises are commonly performed either with a fixed scapular position—intended to limit compensatory movements and emphasize prime movers such as the latissimus dorsi—or with free scapular motion to allow a more natural movement pattern, the neuromuscular implications of these two approaches remain insufficiently characterized [30,31,32]. This has led to an anecdotal perception of major different prime movers involvement between the two variations. Importantly, fixed and free scapular conditions differ not only in movement execution but also in the contraction strategies required for scapular stabilization. Constraining scapular motion predominantly involves sustained isometric activation of stabilizing muscles, whereas a free scapular condition requires dynamic concentric and eccentric actions. These differences may alter both muscle excitation patterns and overall neuromuscular coordination [33,34,35,36,37]. Moreover, neuromuscular demands are influenced by how external loads are managed across movement phases. Accelerating a given load during dynamic contractions increases force requirements during the concentric phase, whereas the same load imposes lower mechanical demands during the eccentric phase [4,38,39]. However, how these phase-dependent demands interact with scapular constraints during horizontal pulling exercises remains largely unexplored. From a biomechanical perspective, scapular position and motion play a critical role in determining shoulder muscle function during horizontal pulling exercises [40,41]. During rowing tasks, coordinated scapulothoracic motion—including scapular retraction, external rotation, and posterior tilt—contributes to optimizing glenohumeral alignment and force transmission [40]. Muscles such as the serratus anterior and trapezius act synergistically to control these movements, while alterations in scapular kinematics can modify muscle length, moment arms, and regional loading within both stabilizing and prime mover muscles [41,42]. Constraining scapular motion may therefore alter not only the magnitude of muscle excitation, but also its spatial distribution across different muscle regions, providing a biomechanical basis for potential shifts in excitation centroids between free and fixed scapular conditions [41,43].

In the recent years, surface electromyography has advanced markedly with the development of high-density sEMG (HD-sEMG), enabling spatial mapping of muscle excitation rather than relying solely on traditional amplitude-based analyses [44,45,46,47]. This technique offers a more detailed characterization of muscle involvement during resistance exercise by assessing the spatial distribution of excitation across the muscle surface, which can be summarized using excitation centroids along the mediolateral and craniocaudal directions [44]. HD-sEMG has already been applied to describe spatial excitation differences within the hamstrings across exercise variations [48], to compare excitation patterns during distinct lat pull-down [20], bench press [11], overhead press techniques [49], and seated row with different grip widths [28]. Nevertheless, the combined influence of scapular constraint and contraction phase on spatial excitation patterns during horizontal pulling exercises has not yet been systematically investigated.

Beyond sport performance contexts, similar neuromuscular variables have been investigated in clinical and rehabilitation settings. Surface electromyography has been used to characterize altered muscle activation patterns in individuals with shoulder pain or dysfunction, particularly in relation to scapular stabilizing muscles and upper-back musculature. Previous studies have reported differences in activation timing, amplitude, and coordination of the trapezius and surrounding muscles in populations with shoulder impingement or scapular dyskinesis, highlighting the clinical relevance of assessing neuromuscular control during upper-limb tasks [31,41,42]. More recently, spatial and phase-specific analyses of muscle activation have been proposed as valuable tools to better understand altered motor strategies in both pathological and rehabilitative conditions [50,51].

Despite the well-established role of contraction type in shaping acute neuromuscular responses [39], short-term adaptations [52], and long-term training outcomes [53,54], limited evidence is currently available regarding the effects of concentric and eccentric actions on spatial patterns of muscle excitation [15,23]. This gap is particularly evident in the context of horizontal pulling exercises performed under different scapular constraints. Accordingly, the aim of this study was to compare both the amplitude and the spatial distribution of muscle excitation between a seated row performed with a fixed scapular position (fixed-SR) and a free scapular position (free-SR) in resistance-trained men. Muscle excitation was assessed using high-density surface electromyography (HD-sEMG), with analyses conducted separately for the concentric and eccentric phases of the movement across key upper-body muscles. From a theoretical standpoint, scapular position and movement phase represent two independent but potentially interacting determinants of neuromuscular demand. Scapular constraint is expected to primarily influence stabilizing requirements and muscle length conditions, whereas contraction phase modulates force production strategies and mechanical tension. Consequently, both main effects of scapular position and contraction phase were anticipated, together with a possible interaction whereby the impact of scapular constraint would differ between concentric and eccentric actions. Isometric and dynamic contractions are known to impose distinct mechanical and neural demands, with isometric stabilization often associated with sustained motor unit activity and high internal stabilizing tension, whereas dynamic actions involve phase-specific force production strategies and changes in muscle length. These differences suggest that scapular constraint may not reduce overall muscle excitation per se, but rather alter how neuromuscular demands are distributed across muscles and movement phases [39,55].

Based on the distinct stabilization strategies required by the two conditions, it was hypothesized that scapular constraint during the fixed-SR would alter the neuromuscular demands placed on scapular stabilizers and prime movers in a phase-dependent manner. Specifically, we expected the fixed-SR to be characterized by greater stabilizing demands on muscles such as the middle trapezius and posterior deltoid, particularly during the eccentric phase, whereas dynamic scapular motion in the free-SR was expected to redistribute force production across movement phases. Accordingly, differences were anticipated in both the magnitude and spatial distribution of muscle excitation between conditions and contraction phases.

## 2. Materials and Methods

### 2.1. Study Design

The study employed a randomized cross-over protocol with a repeated-measures, within-participant structure, consistent with earlier works in this area [15,20,28,49]. Each participant attended three laboratory visits. During the initial visit, individuals were introduced to both the fixed scapular position (fixed-SR) and free scapular position seated-row (free-SR) techniques and the optimal placement of the electrode grids for each muscle was determined. The second visit involved assessing the 8-repetition maximum (8-RM) for the two SR conditions, with the testing order counterbalanced across participants [20,28]. The final visit began with the collection of maximal voluntary isometric contractions for all muscles, followed by a minimum of 10 min of passive rest. Thereafter, surface electromyography (sEMG) signals were recorded while participants performed a controlled set of each SR variation using their respective 8-RM load, completing four repetitions. The limited number of repetitions and the rest intervals provided were intended to minimize the influence of neuromuscular fatigue on the recorded signals. An 8-RM load was selected to reflect a commonly adopted intensity in resistance training practice while ensuring sufficient mechanical demand to elicit clear neuromuscular responses [56]. Although submaximal loads (e.g., 60–70% of one-repetition maximum) are frequently used in electromyographic studies to minimize fatigue, higher relative loads have also been shown to provide reliable sEMG measurements when the number of repetitions is limited and adequate recovery is provided [6,57]. In the present study, only four repetitions were performed per condition, well below the maximal repetition capacity associated with the 8-RM load, and a minimum rest period of 3 min was allowed between conditions. This approach was adopted to minimize the accumulation of neuromuscular fatigue and to focus the analysis on steady-state repetitions rather than on fatigue-induced alterations in muscle excitation. A minimum recovery interval of 72 h was imposed between experimental sessions. During the study period, participants were instructed to refrain from engaging in any other resistance training activities.

### 2.2. Participants

Fourteen resistance-trained men volunteered to participate in the investigation. All participants had a minimum of two years of continuous resistance-training experience and were familiar with multi-joint upper-body resistance exercises. Participants were eligible for inclusion if they were resistance-trained men aged between 18 and 35 years and had not experienced any injuries to the shoulder complex, upper extremities, or spinal region within the six months preceding the study. Exclusion criteria included the presence of current musculoskeletal pain, neurological disorders, or any medical condition that could interfere with the execution of upper-body resistance exercises. Participants were also instructed to refrain from consuming caffeine, alcohol, or other stimulants during the 24 h preceding each experimental session. The study protocol received approval from the Ethics Committee of the University of Milan (Milan, Italy; protocol no. CE 11/23; approved on 9 February 2023). All procedures adhered to the principles outlined in the Declaration of Helsinki (1964 and later revisions). The study involved healthy participants performing standardized resistance exercises and did not include any clinical or therapeutic intervention; therefore, registration as a clinical trial was not required. Before taking part in the investigation, participants were informed in detail about the study aims and experimental procedures and provided written informed consent. They were also informed of their right to withdraw from the study at any time.

No a priori sample size calculation was performed. The sample size was determined based on previous studies employing high-density surface electromyography to investigate neuromuscular excitation during resistance exercises, which have reported meaningful effects using comparable sample sizes [11,20,28,48,49]. Moreover, the randomized cross-over design adopted in the present study increased statistical power by allowing each participant to serve as their own control. In line with recent methodological perspectives, studies characterized by precise physiological measurements and within-subject designs may yield robust and informative results even with relatively small samples, particularly when the primary aim is mechanistic rather than predictive or epidemiological [58].

### 2.3. Exercise Technique

**Figure 1:**
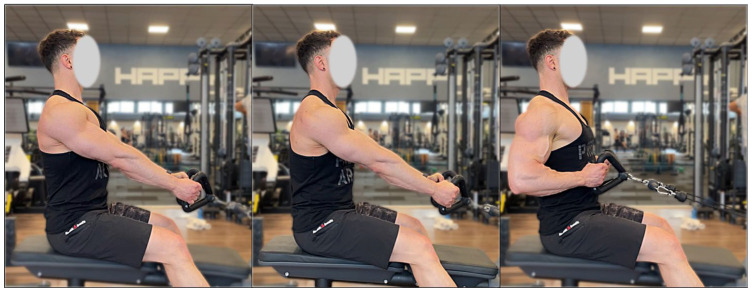
Presentation of the technique for each exercise. From the left: the starting position of the fixed-SR, the starting position of the free-SR, the end position.

Participants executed both the fixed- and the free-SR on a commercial weight-stack seated row machine (Technogym, Cesena, Italy). Participants were seated without trunk or chest support, while foot placement could be adjusted to achieve a comfortable and stable starting position. No mechanical constraints were imposed on scapular motion by the machine itself. The technical execution of each variation is shown in Figure 1. A standard triangle handle (Technogym, Cesena, Italy) was used for both conditions. For the fixed-SR, the initial setup involved fully extended elbows, a slightly flexed knee position, an upright torso, retracted shoulders, and abducted scapulae. Starting from this posture, the concentric phase involved a coordinated movement characterized by shoulder extension accompanied by elbow flexion until the handle reached the abdominal region. A researcher supervised each attempt to ensure that participants maintained the required technique. In the free-SR, participants began with straight elbows, a neutral torso, and slightly flexed knees; however, the shoulders were protracted and the scapulae adducted at the start. The pull was initiated by actively adducting the scapulae, followed by humeral extension and elbow flexion, with the concentric endpoint defined when the handle reached the abdominal target abovementioned. Throughout the eccentric phase, trunk movement was minimized by requiring participants to maintain a stable posture without flexion or extension. At the end of the lowering phase, the load was paused isometrically for approximately 0.5 s before beginning the next concentric action. The full movement was executed according to established resistance-training guidelines [1]. Both SR variations followed an identical repetition tempo: 2 s for the concentric movement, 2 s for the eccentric movement, and a brief isometric hold of roughly 0.5 s [15,20,28,49]. Participants also received continuous visual timing feedback to ensure adherence to the prescribed tempo [20,28]. It should be acknowledged that the fixed-SR and free-SR conditions differed primarily in scapular positioning and movement initiation strategy, while overall humeral alignment and trunk posture were maintained comparable between conditions. Participants were instructed to keep a stable, upright seated posture in both variations, minimizing trunk flexion and extension throughout the movement. In both conditions, the humeral position relative to the torso and the pulling trajectory were similar, with differences in shoulder appearance arising mainly from changes in scapulothoracic configuration rather than from true alterations in shoulder joint alignment. In the free-SR condition, the more anterior appearance of the shoulders reflects the natural anterior translation of the scapulae allowed by unconstrained scapulothoracic motion, whereas in the fixed-SR the scapulae were maintained in a retracted position throughout the movement, requiring sustained stabilization. Although scapular motion can theoretically involve components of rotation and adduction, the fixed handle width and the horizontal nature of the pulling task substantially limited variability in these degrees of freedom. Consequently, differences between conditions were primarily expressed along the anteroposterior axis through scapular protraction and retraction, which represented the main focus of the present comparison.

### 2.4. Muscle Excitation Detection

High-density surface EMG signals were acquired using semi-reusable two-dimensional electrode grids arranged in a 13 × 5 configuration (GR08MM1305; 8 mm interelectrode distance, OT Bioelettronica, Turin, Italy) during both seated-row conditions. Signals were recorded in monopolar mode and subsequently processed offline. Recordings were obtained from the dominant side, targeting the upper, middle, and lower trapezius, latissimus dorsi, lateral and posterior deltoid, biceps brachii, triceps brachii, and erector spinae muscles. For the lateral deltoid, posterior deltoid, biceps brachii, triceps brachii, and erector spinae, grids were oriented parallel to the estimated muscle-fiber direction [59]. Conversely, for the trapezius subdivisions and latissimus dorsi, the grids were positioned perpendicularly to the fiber orientation [44]. The innervation zone was not considered when positioning grids on the upper, middle, and lower trapezius, latissimus dorsi, or erector spinae based on the “Atlas of Muscle Innervation Zones” [59]. For the lateral and posterior deltoid, biceps brachii, and triceps brachii, grid placement took the innervation region into account following prior recommendations [20,28,60]. In these cases, care was taken to minimize signal contamination from adjacent musculature [44].

The placement of each grid followed established anatomical guidelines. For the upper trapezius, the grid was positioned approximately 2 cm lateral to the C7 spinous process [59]. The middle trapezius grid was located roughly 2 cm lateral to the thoracic spine and directly below the upper trapezius placement [59]. For the lower trapezius, the grid was applied 2 cm lateral to the spine and superior to the T12 spinous process [59]. The latissimus dorsi grid was placed 2 cm inferior to the scapular angle and aligned parallel to the spinal column [59]. For the lateral deltoid, the grid was centered midway between the acromion and the lateral epicondyle, positioned above the deltoid tuberosity [59]. The posterior deltoid grid was placed on the upper portion of the muscle, approximately 2 cm medial to the posterolateral border of the acromion. The biceps brachii grid was positioned on the proximal segment of the long head, aligned along the line between the acromion and the distal tendon insertion [59]. For the long head of the triceps brachii, the grid was applied at one-third of the distance from the acromion to the medial humeral epicondyle [59]. The erector spinae grid was positioned 2 cm lateral to the lumbar spine over the L5 vertebral level [59]. A conductive paste (AC cream, Spes Medica, Genoa, Italy) was applied to each grid cavity to ensure stable skin–electrode contact. Skin preparation consisted of shaving and gentle abrasion using an abrasive paste (Nuprep, Weaver and Company, Aurora, CO, USA). Signals were recorded in monopolar configuration at 2048 Hz with an amplification gain of 200 [61], using a multichannel EMG system (EMG-USB2+, OT Bioelettronica, Turin, Italy) [62]. The wrist served as the ground site, whereas reference electrodes were positioned on the acromion for the HD-sEMG grids. After electrode placement, participants completed maximum voluntary isometric contractions (MVICs) for each muscle using a fixed cable attachment positioned to maintain a 90° elbow angle. Contractions from both seated-row variations were collected in randomized order [28,63]. Three attempts per muscle were performed, each lasting 5 s, with 3 min of rest provided between efforts [20,28]. Standardized verbal encouragement was delivered to facilitate maximal effort. Following a passive rest of 10 min, participants executed a non-exhaustive set of both SR variations (order randomized). Loads corresponded to each participant’s predetermined 8-RM, and a 3 min inter-set rest interval was provided. Four repetitions were performed per condition to reduce cumulative fatigue and ensure consistent technique. The repetition cadence was regulated using a metronome, adopting a 2 s concentric phase, a 2 s eccentric phase, and an isometric pause of roughly 0.5 s. An investigator monitored all repetitions to ensure adherence to the prescribed tempo and movement execution.

### 2.5. Muscle Excitation Centroid

High-density electrode grids allow for the examination of how muscle excitation is distributed across the grid surface. To characterize this spatial pattern, RMS values from each channel were used to compute the barycenter of excitation along the horizontal (x-axis) and vertical (y-axis) directions, expressed in millimeters relative to the grid coordinate system. This measure, often referred to as the central locus of excitation, provides an index of the spatial distribution of sEMG amplitude [64]. The resulting centroid—representing the weighted average position of sEMG activity across all grid rows and columns—was determined for the upper, middle, and lower trapezius; latissimus dorsi; lateral and posterior deltoid; biceps brachii; triceps brachii; and erector spinae muscles [65].

### 2.6. Data Analysis

sEMG signals were acquired in monopolar configuration with an amplification gain of 200 [61] and digitized at 2048 Hz using a 12-bit A/D converter operating within a 5 V range. The raw signals were processed using a 20–400 Hz band-pass filter [62]. Muscle excitation in the time domain was quantified through the root mean square (RMS). For the maximal voluntary isometric contractions, a 1 s epoch corresponding to the highest stable portion of the signal was analyzed, and for each muscle the greater value obtained between the two seated-row conditions was retained. During the exercise trials, RMS values were computed for the central second of both the concentric and eccentric phases. To synchronize the sEMG recordings with the movement phases of each repetition, a digital video camera (iPhone 12; 12 MP; 1080 p resolution at 60 frames·s^−1^; Apple Inc., Cupertino, CA, USA) was mounted on a tripod and used to identify the onset and termination of the concentric and eccentric phases during offline analysis This video-based synchronization approach has been widely adopted in previous surface electromyography studies investigating resistance training exercises, where phase identification is primarily aimed at ensuring consistent comparisons across conditions rather than precise kinematic modeling [11,13,14,20,28,49]. Although no motion sensors, force transducers, or joint angle measurements were employed, the same segmentation procedure was consistently applied across all repetitions, muscles, phases, and experimental conditions, thereby minimizing the likelihood of systematic bias in between-condition comparisons. To further reduce the influence of transient artifacts and potential timing inaccuracies, RMS values were calculated from the central portion of each movement phase. To ensure consistent execution and avoid transient artifacts, the first repetition of every set was omitted from the analysis [66]. All RMS values obtained during exercise were subsequently normalized to the peak MVIC for the corresponding muscle (nRMS), following previous procedures [15,20,24,28].

**Figure 2:**
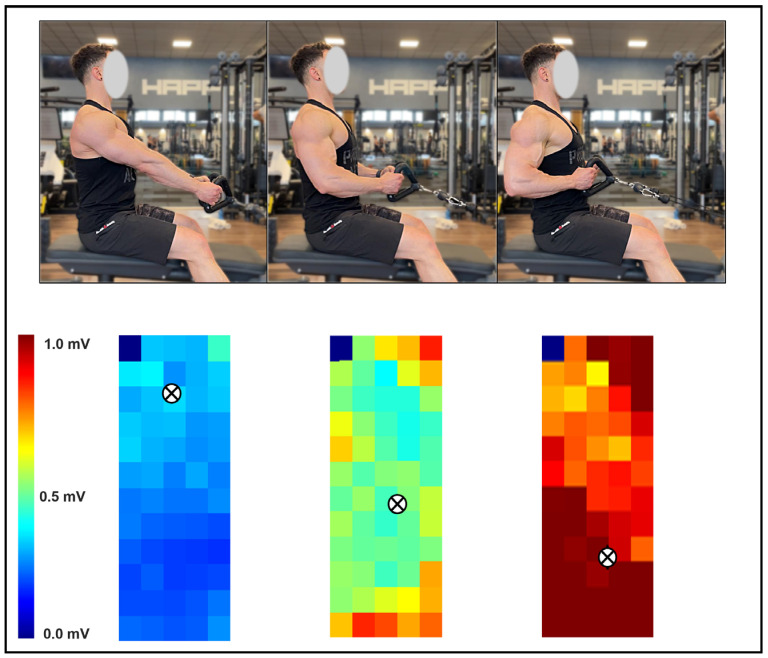
Illustrative example of the spatial excitation pattern of the latissimus dorsi recorded with HD-sEMG during the fixed-scapular-position seated row. Three movement positions are shown in the upper panel, with their corresponding excitation maps displayed below. The centroid of excitation is denoted by the symbol “⊗”.

Color maps of muscle excitation were created in MATLAB R2023B (The MathWorks, Natick, MA, USA) using the RMS amplitudes from all 64 channels of each grid (Figure 2). To generate these maps, monopolar signals were first band-pass filtered (20–400 Hz) and then converted into either 12 or 4 single-differential channels depending on the relationship between the muscle-fiber orientation and the grid layout [11]. RMS values were then calculated for each differential signal. Only channels classified as “active”—those with RMS amplitude exceeding 70% of the maximum RMS recorded across the matrix—were included in subsequent analyses [11]. This threshold was selected because it reliably isolates electrodes positioned above the most excited muscular regions [67]. For each grid, the number of active channels and their interquartile range were calculated to assess the spatial dispersion of muscle excitation. The spatial barycenter of the active channels was also computed, providing a weighted coordinate that indicates where RMS amplitude was most prominent along the cranio-caudal and medio-lateral axes. Electrode positions were converted into x- and y-coordinates expressed in millimeters, allowing the centroid to be localized precisely within the electrode matrix [20,28]. The analysis of both upward and downward movement phases focused on the central second of each phase to avoid transition artifacts.

### 2.7. Statistical Analysis

Data distribution was assessed for normality using the Shapiro–Wilk test. As all variables were normally distributed, parametric statistical procedures were applied and descriptive data are presented as mean (SD). Differences in normalized sEMG amplitude (nRMS) and excitation centroid coordinates (mediolateral and craniocaudal axes) were examined using two-way repeated-measures analyses of variance (ANOVA), with Condition (fixed-SR vs. free-SR) and Phase (concentric vs. eccentric) included as within-subject factors and analyses performed separately for each muscle. When significant main effects or interactions were detected, post hoc pairwise comparisons were conducted using Bonferroni-adjusted contrasts. Effect size estimates were reported using partial eta-squared (ηp^2^) for ANOVA models and Cohen’s d for pairwise comparisons. Partial eta-squared values were interpreted according to commonly used thresholds in the context of repeated-measures designs, as small (0.01), moderate (0.06), and large (0.14). For Cohen’s d, effect sizes were interpreted using group-difference-specific thresholds recommended in the physiotherapy and rehabilitation literature, where values of 0.1, 0.4, and 0.8 represent small, moderate, and large effects, respectively [68]. These thresholds were adopted to provide a more domain-specific and clinically meaningful interpretation of the magnitude of between-condition differences. All statistical analyses were performed using R software (version 4.5.1; R Core Team, Vienna, Austria), with the tidyverse, afex, and emmeans packages. Statistical significance was set at α = 0.05.

## 3. Results

The sociodemographic and anthropometric characteristics of the participants are summarized in Table 1. Fourteen resistance-trained men were included in the analysis. Participants were young adults with several years of resistance-training experience and showed relatively homogeneous anthropometric characteristics in terms of age, stature, and body mass.

**Figure 3:**
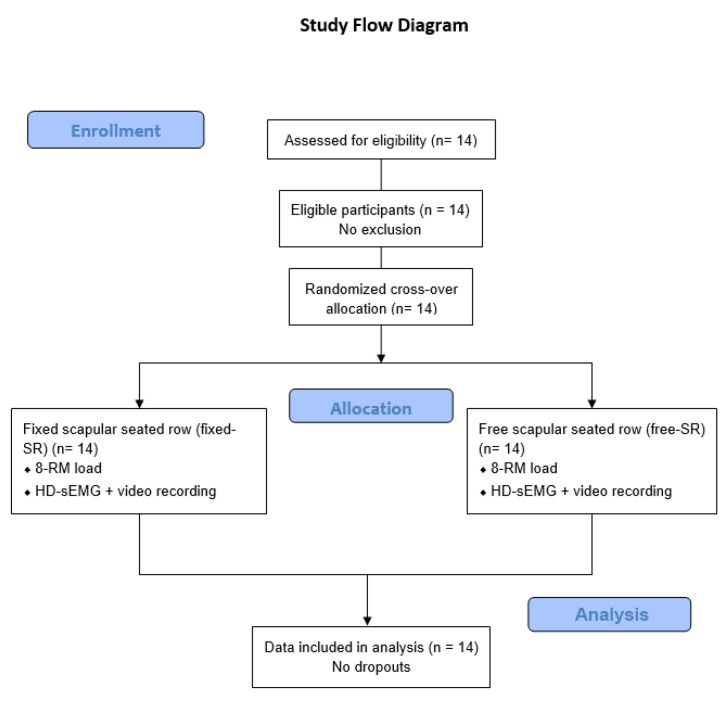
Flow diagram of the study design and participant progression through the experimental protocol.

A schematic overview of the study design and participant progression through the experimental protocol is shown in Figure 3.

The average 8-RM load was 49.9 ± 6.1 kg for the free-SR and 50.3 ± 5.7 kg for the fixed-SR (*p* > 0.05).

**Figure 4:**
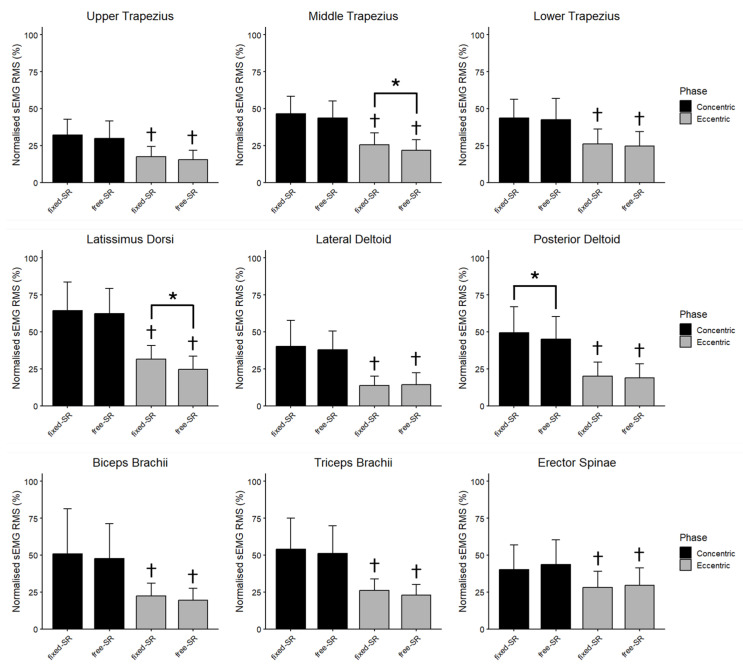
The mean (SD) of the normalized root mean square (nRMS) recorded during the concentric and the eccentric phase of fixed scapular position seated row (fixed-SR) and free scapular position seated row (free-SR) is shown for each muscle. Besides fixed vs. free seated row differences, nRMS was greater during the concentric than the eccentric phase for all muscles. *: *p* < 0.05 vs. free-SR. †: *p* < 0.05 vs. eccentric phase.

Figure 4 displays the global nRMS recorded from all muscles during the concentric and eccentric phases in the free- and fixed-SR. No interaction between exercise and phase was observed for the nRMS in the upper trapezius (F = 0.003, *p* = 0.956, ηp^2^ = 0.000), middle trapezius (F = 0.607, *p* = 0.450, ηp^2^ = 0.045), lower trapezius (F = 0.043, *p* = 0.838, ηp^2^ = 0.003), latissimus dorsi (F = 2.607, *p* = 0.130, ηp^2^ = 0.167), lateral deltoid (F = 1.277, *p* = 0.279, ηp^2^ = 0.089), posterior deltoid (F = 2.050, *p* = 0.176, ηp^2^ = 0.136), biceps brachii (F = 0.025, *p* = 0.876, ηp^2^ = 0.002), triceps brachii (F = 0.013, *p* = 0.911, ηp^2^ = 0.001) and erector spinae muscles (F = 0.856, *p* = 0.372, ηp^2^ = 0.062). During the concentric phase, the nRMS was higher in the fixed- than the free-SR in the posterior deltoid (4.37%, 0.80% to 7.94%; ES = 0.66, 0.10 to 1.21), while the upper, middle, lower trapezius, latissimus dorsi, lateral deltoid, biceps brachii, triceps brachii, and erector spinae muscles had similar excitation (*p* > 0.05). In the eccentric phase, the nRMS was higher in the fixed- than in the free-SR in the middle trapezius (3.72%, 0.71% to 6.72%; ES = 0.67, 0.11 to 1.22), and in the latissimus dorsi (6.83%, 2.47% to 11.20%; ES = 0.85, 0.25 to 1.43), while the upper, lower trapezius, lateral deltoid, posterior deltoid, biceps brachii, triceps brachii, and erector spinae muscles had similar excitation (*p* > 0.05).

**Figure 5:**
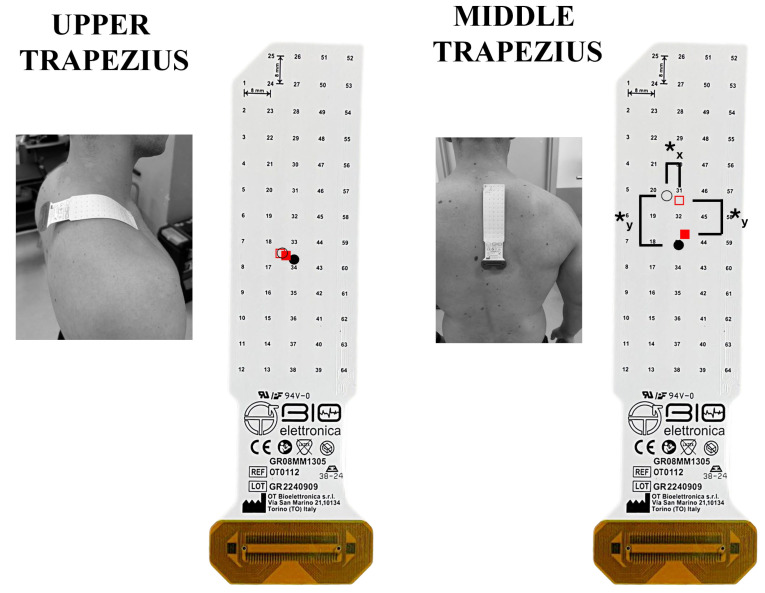

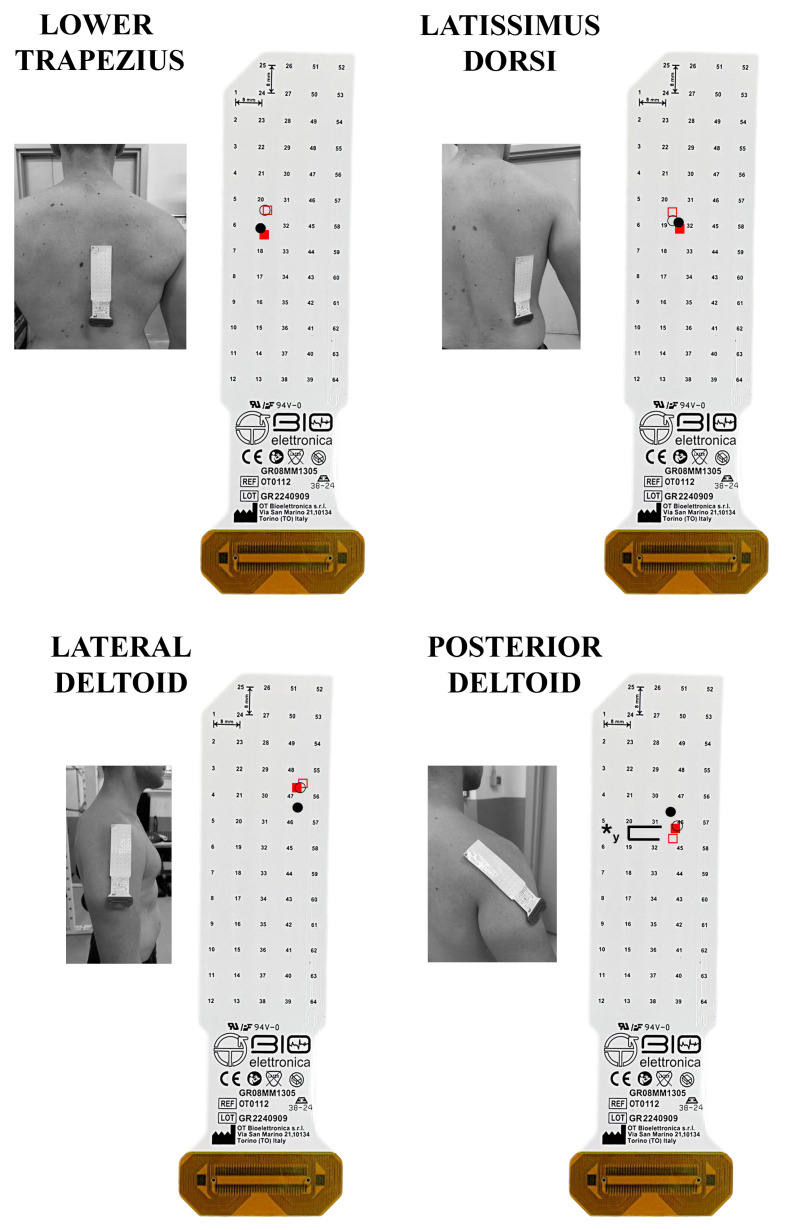

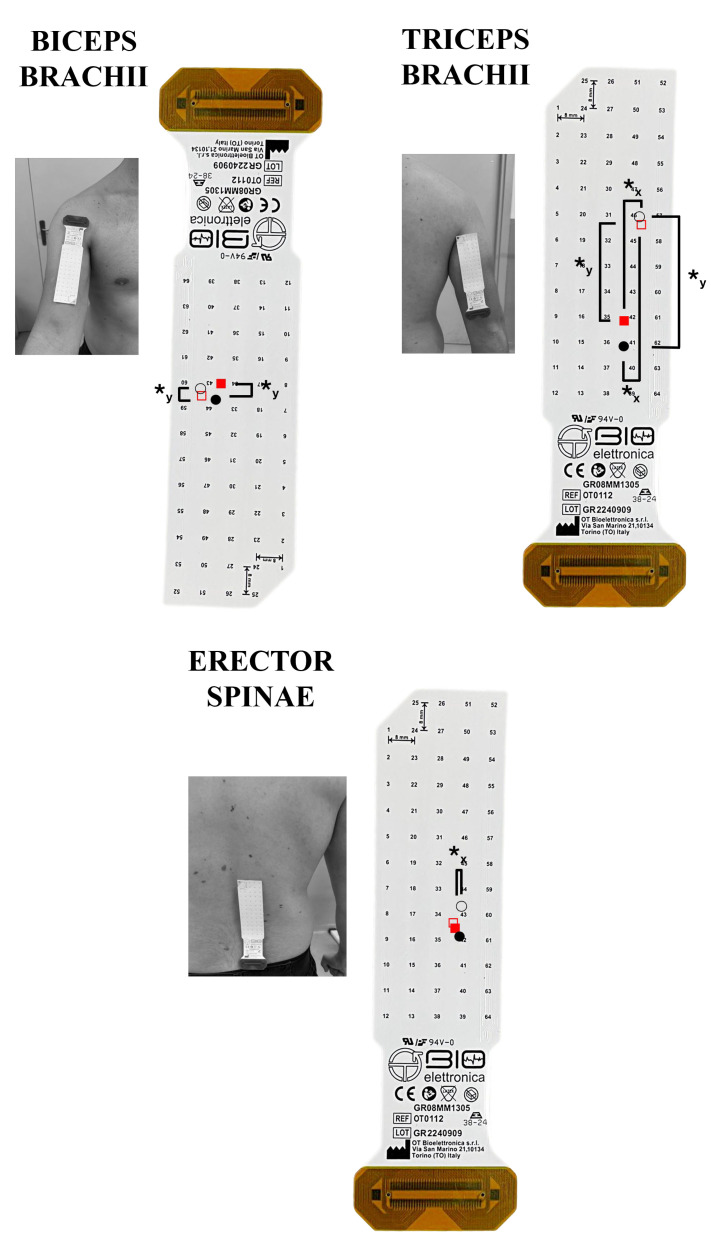
The spatial muscle excitation for the muscles analyzed is shown. The grids are visualized as positioned on each muscle. The upward and downward direction indicates a cranial and caudal shift on the vertical plane, respectively; the rightward and leftward shifts indicate a lateral and medial shift on the horizontal plane, respectively. The fixed scapular position seated row (fixed-SR) is represented graphically by fill red squares (◼) for the concentric and empty red squares (◻) for the eccentric phase. The free scapular position seated row (free-SR) is represented graphically by fill circles (⚫) for the concentric and empty circles (**⭘**) for the eccentric phase. ✱y: *p* < 0.05 comparing the centroid on the vertical y-axis. ✱x: *p* < 0.05 comparing the centroid on the horizontal x-axis.

Figure 5 illustrates the average horizontal and vertical coordinates of the centroid for each muscle during the concentric and eccentric phases of both the fixed- and the free-SR. An interaction between exercise and phase was observed for the horizontal coordinates in the upper trapezius (F = 0.025, *p* = 0.002, ηp^2^ = 0.002), biceps brachii (F = 11.402, *p* = 0.005, ηp^2^ = 0.467), and erector spinae (F = 9.289, *p* = 0.009, ηp^2^ = 0.417). In contrast, no interaction was identified in the middle trapezius (F = 2.111, *p* = 0.170, ηp^2^ = 0.140), lower trapezius (F = 1.215, *p* = 0.290, ηp^2^ = 0.085), latissimus dorsi (F = 0.201, *p* = 0.662, ηp^2^ = 0.015), lateral deltoid (F = 0.711, *p* = 0.414, ηp^2^ = 0.052), posterior deltoid (F = 0.121, *p* = 0.733, ηp^2^ = 0.009), triceps brachii (F = 2.957, *p* = 0.109, ηp^2^ = 0.185). During the concentric phase, no medial-lateral differences in the centroid were found for the upper, middle, lower trapezius, latissimus dorsi, lateral deltoid, posterior deltoid, biceps brachii, triceps brachii and erector spinae muscles (*p* > 0.05). During the eccentric phase, the centroid was positioned more laterally in the fixed- vs. the free-SR in the middle trapezius (0.41%, 0.09% to 0.72%; ES = 0.54, 0.01 to 1.07), while no medial-lateral differences in the centroid were observed for the upper trapezius, lower trapezius, latissimus dorsi, lateral deltoid, posterior deltoid, biceps brachii, triceps brachii, and erector spinae muscles (*p* > 0.05). The centroid was positioned more medially in the triceps brachii during the concentric phase compared to the eccentric phase of both the fixed and free-SR (2.98%, 1.50% to 4.46%, ES = 1.09, 0.44 to 1.73; 4.16%, 2.61% to 5.71%, ES = 1.01, 0.37 to 1.62). In addition, the centroid was positioned more medially in the erector spinae (1.04%, 0.17% to 1.91%, ES = 0.11, −0.38 to 0.61) during the concentric phase compared to the eccentric phase of the free-SR. No additional between-phase differences were observed (*p* > 0.05).

An interaction between exercise and the phase was observed for the vertical coordinates in the posterior deltoid (F = 7.587, *p* = 0.016, ηp^2^ = 0.369), conversely no interaction between exercise and phase was observed for the vertical axis in the upper trapezius (F = 0.541, *p* = 0.596, ηp^2^ = 0.083), middle trapezius (F = 0.254, *p* = 0.623, ηp^2^ = 0.019), lower trapezius (F = 0.133, *p* = 0.722, ηp^2^ = 0.010), latissimus dorsi (F = 0.289, *p* = 0.600, ηp^2^ = 0.022), lateral deltoid (F = 1.156, *p* = 0.302, ηp^2^ = 0.082), biceps brachii (F = 0.249, *p* = 0.626, ηp^2^ = 0.019), triceps brachii (F = 0.298, *p* = 0.595, ηp^2^ = 0.022) and erector spinae muscles (F = 0.265, *p* = 0.771, ηp^2^ = 0.042). During the concentric phase, no cranio-caudal differences in the centroid were found for the upper trapezius, middle trapezius, lower trapezius, latissimus dorsi, lateral deltoid, posterior deltoid, biceps brachii, triceps brachii, and erector spinae muscles (*p* > 0.05). During the eccentric phase, the centroid was positioned more caudally in the fixed- vs. the free-SR in the posterior deltoid (0.16%, 0.03% to 0.30%, ES = 0.22, −0.28 to 0.72), while no cranio-caudal differences in the centroid were observed for the upper, middle, lower trapezius, latissimus dorsi, lateral deltoid, biceps brachii, triceps brachii, and erector spinae muscles (*p* > 0.05). Additionally, the centroid was more caudal in the concentric phase compared to the eccentric phase of both the fixed and free-SR in the middle trapezius (1.21%, 0.39% to 2.03%; ES = 0.52, −0.14 to 1.05; 1.56%, 0.17% to 2.96%; ES = 0.61, 0.05 to 1.14), biceps brachii (0.38%, 0.17% to 0.58%; ES = 0.22, −0.28 to 0.72; 0.43%, 0.20% to 0.65%; ES = 0.02, −0.47 to 0.51), and triceps brachii (0.46%, 0.25% to 0.67%; ES = 0.62, 0.06 to 1.16; 0.43%, 0.20% to 0.66%; ES = 0.70, 0.13 to 1.24). No further between-phase difference was found (*p* > 0.05).

## 4. Discussion

The present study is the first to investigate the excitation patterns of the primary muscles involved in the seated row when performed with a fixed scapular position (fixed-SR) and a free scapular position (free-SR), using high-density surface EMG (HD-sEMG) and analyzing the concentric and eccentric phases separately. The main outcomes can be summarized as follows. First, both variations were executed with comparable external loads. Second, during the concentric phase, the posterior deltoid exhibited greater excitation in the fixed-SR. In the eccentric phase, higher excitation of the middle trapezius and latissimus dorsi was also observed in the fixed-SR relative to the free-SR. Third, in the medio-lateral direction, the centroid of the middle trapezius was located more laterally in the fixed-SR during the eccentric phase. Finally, in the cranio-caudal direction, the posterior deltoid centroid was positioned more caudally in the fixed-SR during the eccentric phase. Although we hypothesized the isometric contraction during the fixed-SR would turn into a lower excitation compared to the concentric phase and higher excitation compared to the eccentric phase of the free-SR, only the latter was confirmed.

Before interpreting the excitation patterns observed in the present study, several contextual points should be addressed. Importantly, the absolute load used in the fixed-SR and free-SR was comparable, indicating that load magnitude did not influence the differences found in sEMG amplitude. Although higher external loads typically produce greater RMS values [8], variations in technique can alter joint mechanics in ways that either enhance or limit the capacity to lift heavier loads [7]. In the current investigation, however, this factor did not introduce meaningful differences between conditions. This equivalence in loading is essential when considering the relative involvement of each primary muscle. Within this context, it is likely that changes in scapular positioning represented the main factor driving differences in muscle excitation. The altered scapular orientation may position the muscles at distinct operating lengths, and such length-related shifts are known to influence the amplitude of sEMG signals [6].

During the concentric phase, the posterior deltoid exhibited greater excitation in the fixed-SR compared with the free-SR. This difference is likely attributable to the altered initiation strategy imposed by scapular constraint. In the fixed-SR condition, the scapulae start in a retracted position, limiting the contribution of dynamic scapular motion at the onset of the pull. Consequently, movement initiation relies more heavily on humeral extension rather than on scapular retraction, increasing the mechanical demand placed on the posterior deltoid during the initial portion of the concentric phase. In contrast, in the free-SR condition, active scapular retraction contributes to movement initiation, allowing a more distributed sharing of the load across scapular and glenohumeral muscles. This difference in initiation strategy likely explains the greater posterior deltoid excitation observed in the fixed-SR [30]. This interpretation is consistent with biomechanical evidence indicating that the posterior deltoid contributes substantially to humeral extension torque during horizontal pulling tasks, particularly when the contribution of scapular retractors is reduced [69,70].

During the eccentric phase, both the middle trapezius and the latissimus dorsi exhibited greater excitation in the fixed-SR compared with the free-SR. This finding can be interpreted as a consequence of the increased stabilizing demands imposed by scapular constraint. In the fixed-SR condition, the scapulae remain adducted throughout the movement, requiring sustained activation of the scapular stabilizers to resist passive protraction during load lowering. As a result, the middle trapezius is required to maintain a quasi-isometric stabilizing role, while the latissimus dorsi contributes more substantially to controlling humeral flexion during the eccentric action. Previous biomechanical and electromyographic studies have demonstrated that the latissimus dorsi plays a major role in regulating humeral motion and shoulder joint loading during eccentric phases of pulling movements, especially when stabilizing demands at the scapulothoracic joint are increased [69,71]. From a joint-mechanics perspective, this role is consistent with the primary function of the middle trapezius in generating scapular retraction moments and resisting protraction forces during horizontal pulling tasks, particularly when scapular translation is mechanically constrained [30,69]. Together, these demands likely explain the higher sEMG amplitude observed for both muscles in the fixed-SR during the eccentric phase [20,30]. Beyond the biomechanical interpretation, the observed differences in muscle excitation can be discussed within a broader neuromuscular and physiological framework. Surface EMG amplitude primarily reflects neural drive to the muscle, integrating motor unit recruitment and discharge rate [6,72]. Therefore, the greater excitation observed in specific muscles during the fixed-SR condition likely indicates an increased neural demand to maintain force output under constrained scapular mechanics [72].

From a physiological perspective, sustained or quasi-isometric activation, such as that required of the middle trapezius during the fixed-SR, is associated with prolonged motor unit activity and increased local metabolic demand [3,72]. Over time, such activation patterns may contribute to region-specific adaptations in muscle architecture, including changes in fascicle behavior and connective tissue loading. Similarly, the increased eccentric excitation of the latissimus dorsi and posterior deltoid may reflect enhanced mechanical tension during lengthening contractions, a stimulus known to play a central role in promoting structural remodeling of skeletal muscle tissue [3,55]. Although the present study did not directly assess morphological adaptations, the spatial excitation patterns identified here provide mechanistic insight into how different exercise techniques may bias neuromuscular loading across muscle regions.

When interpreting spatial excitation metrics derived from HD-sEMG, it is essential to consider the orientation of the electrode grid relative to the underlying muscle fiber direction. The physiological meaning of excitation centroid displacement depends on whether the analyzed axis is aligned parallel or transverse to the main fiber orientation [44]. Displacements along axes parallel to muscle fibers are more closely associated with factors such as changes in motor unit recruitment distribution or shifts in the innervation zone during dynamic contractions, whereas displacements along transverse axes primarily reflect regional differences in excitation across distinct muscle portions [44,73]. Accordingly, centroid position does not necessarily correspond to anatomical reference planes, as it is defined relative to the electrode matrix rather than to the muscle’s anatomical orientation. Importantly, the alignment between electrode axes and muscle architecture differs across muscles. For example, in the middle trapezius the mediolateral axis is approximately parallel to the fiber direction, whereas in the lateral deltoid it is largely transverse. Therefore, centroid shifts must be interpreted in a muscle-specific manner. Finally, although HD-sEMG allows spatially informed assessment of muscle excitation, the analyzed region is limited to the portion of the muscle covered by the electrode grid, and centroid metrics should be interpreted within this constraint [44]. Comparing the fixed- vs. the free-SR, the centroid of the middle trapezius was more lateral during the eccentric phase. Because the electrode matrix over the middle trapezius was aligned with the mediolateral orientation of its fibers, the lateral centroid displacement observed in the fixed-SR during the eccentric phase can be interpreted as a greater relative contribution of the lateral portion of the muscle, as inferred from the spatial distribution of HD-sEMG excitation [64]. It should be noted, however, that excitation centroid displacement represents an indirect indicator of regional muscle involvement and does not allow direct quantification of force production or localized mechanical loading within the muscle. When scapular motion is constrained, the lateral fascicles—located near the spine of the scapula—carry a larger share of the eccentric braking demand as the humerus moves forward, resulting in increased excitation in this region [64]. This shift in the distribution of excitation moves the centroid laterally, whereas in the free-SR the natural scapular protraction allows a more balanced recruitment between medial and lateral fibers, yielding a stable centroid position.

Comparing the fixed- vs. the free-SR, the centroid of the posterior deltoid was more caudal during the eccentric phase. Because the matrix was aligned with the posterior deltoid fibers, the caudal displacement of the centroid observed in the fixed-SR during the eccentric phase is best interpreted as a greater relative contribution of the distal portion of the muscle, based on changes in the spatial distribution of HD-sEMG excitation [64]. As such, these spatial findings should be interpreted as reflecting relative shifts in neuromuscular excitation rather than definitive evidence of localized muscle force or structural adaptations. Constraining scapular motion increases the mechanical demand on the distal fascicles as the humerus moves into flexion, leading to a more pronounced recruitment in the caudal region of the muscle. This enhanced distal recruitment shifts the centroid caudally [64], whereas in the free-SR the scapulohumeral motion distributes the load more evenly along the fascicle direction, resulting in a more stable centroid position.

From a clinical and applied perspective, understanding how scapular constraints influence muscle excitation has relevant implications for both rehabilitation and strength training practice. Scapular control exercises are commonly prescribed in the management of shoulder disorders, particularly in populations with altered scapulohumeral rhythm or shoulder pain [40,41]. The present findings suggest that constraining scapular motion during horizontal pulling tasks increases the stabilizing demand placed on the middle trapezius, latissimus dorsi, and posterior deltoid, especially during eccentric actions. This information may assist clinicians and practitioners in selecting exercise variations that selectively emphasize scapular stabilizers or posterior shoulder musculature, depending on the rehabilitation or training goal [42]. Conversely, allowing free scapular motion may distribute neuromuscular demands more evenly across muscles, potentially reducing localized loading in individuals sensitive to excessive scapular or glenohumeral stress [40,41].

The present study has some limitations that should be acknowledged. First, although HD-sEMG provides detailed information on the spatial distribution of muscle excitation, it does not allow direct inference about muscle force production or long-term morphological adaptations. Therefore, the present findings should be interpreted as reflecting acute neuromuscular demands rather than training-induced changes in muscle structure. Second, excitation patterns were assessed only at a single load corresponding to the 8-RM, and different loading schemes or movement velocities may yield different neuromuscular responses. Third, the HD-sEMG grids covered only a portion of each muscle, and spatial metrics therefore represent excitation patterns within the sampled regions rather than across the entire muscle volume. Fourth, although participants were carefully instructed and visually supervised to maintain a stable trunk posture across conditions, no kinematic measurements were used to objectively quantify trunk or pelvic rotation. While major differences in movement execution were unlikely due to the standardized setup and supervision, subtle inter-individual variations cannot be entirely excluded. Finally, although the sample size was comparable to previous mechanistic studies using HD-sEMG, larger samples would be required to improve generalizability across populations with different training backgrounds or clinical characteristics.

## 5. Conclusions

The present study compared both the amplitude and the spatial distribution of muscle excitation during the seated row performed with a fixed versus a free scapular position, separately analyzing concentric and eccentric phase using HD-EMG. The results show that scapular constraint does not substantially alter overall muscle excitation levels, as both variations elicited comparable excitation of the primary pulling muscles under equivalent external loads. However, the fixed scapular condition increased some neuromuscular demands in specific upper-back muscles, particularly the posterior deltoid during the concentric phase and the middle trapezius and latissimus dorsi during the eccentric phase. Collectively, these findings provide direct evidence that both variations may emphasize different aspects of scapular stabilization and humeral control. From a practical standpoint, fixed-scapular execution may be preferentially employed to increase stabilizing demands on the upper-back musculature and emphasize controlled humeral motion. In contrast, free-scapular execution allows a greater dynamic range of scapular adduction, enabling the active attainment of the initial degrees of adduction and muscle action at longer lengths. However, these kinematic differences do not necessarily translate into greater muscle excitation, suggesting that the primary effects of scapular mobility are related to movement execution rather than excitation magnitude. Accordingly, the choice between fixed and free scapular execution should be guided by specific training or rehabilitation objectives rather than by rigid notions of correct or incorrect technique. Future research should examine whether the acute differences in spatial excitation observed here translate into divergent long-term adaptations in muscle morphology or function.

## Figures and Tables

**Table 1:** Anthropometric and sociodemographic characteristics of the participants. Data are presented as mean ± standard deviation.

Variable	Mean ± SD
Participants (n)	14
Age (years)	25 ± 4
Height (m)	1.74 ± 0.06
Body mass (kg)	76.2 ± 5.7

## Data Availability

The data presented in this study are available on request from the corresponding author.
